# Anterior Cruciate Ligament Mechanical Response to Load in the Setting of Changes to the Medial Meniscus

**DOI:** 10.3390/bioengineering12010074

**Published:** 2025-01-15

**Authors:** Angela Hussain, Muffaddal Madraswala, Jason Koh, Farid Amirouche

**Affiliations:** 1 Department of Orthopaedic Surgery, University of Illinois at Chicago, Chicago, IL 60612-7342, USA; ahussa64@uic.edu (A.H.); mjuze@uic.edu (M.M.); 2 Department of Orthopaedic Surgery, Orthopaedic and Spine Institute, Northshore University Health System, An Affiliate of the Pritzker School of Medicine, University of Chicago, 9669 Kenton Avenue, Skokie, IL 60076, USA; drjasonkoh@gmail.com

**Keywords:** ACL, meniscus, knee ligament, knee stability

## Abstract

The anterior cruciate ligament (ACL) is a major ligament in the knee joint, and its function is crucial for both the movement and stability of the knee. Our research takes a novel approach by investigating the effect of meniscus tears on the ACL, how such tears will impact the stress on the ACL, and its overall compensation in response to the changes in the meniscus. **Hypothesis/Purpose**: This study aims to investigate how the ACL compensates for the change in knee joint stability and contact pressures due to partial horizontal cleavage tears (HCTs) in the meniscus, such as partial meniscectomy and partial transplantation on knee joint stability and contact pressures. We hypothesize that HCTs will increase contact pressures and decrease joint stability, thereby inducing compensatory stress on the anterior cruciate ligament (ACL). **Method**: Seven freshly frozen human cadaveric knees were used in a study to investigate the effects of different meniscal conditions and surgical interventions on the meniscus itself. Four testing scenarios were established: intact knees, knees with partial horizontal cleavage tears (HCTs) of the meniscus, knees with partial meniscectomy, and knees with partial transplantation. Axial loading was applied, and the medial meniscus contact pressures were measured at 0° and 30° of flexion. Additionally, a mathematical 3D finite element model was created to evaluate the behavior of the ACL under different meniscus scenarios, which could not have been measured experimentally. **Results**: ACL contact pressure and stress analysis across various meniscal conditions demonstrated substantial variability. Horizontal cleavage tears (HCTs) resulted in heightened contact pressures and diminished joint stability, as evidenced by increased ACL stress attributed to compensatory mechanisms in the presence of meniscal tears. Conversely, transplantation procedures exhibited a mitigating effect, maintaining joint mechanics closer to intact conditions and minimizing alterations in ACL forces. These trends persisted at 30 degrees of knee flexion, where significant increases in ACL forces were observed in partial and complete HCT conditions. **Conclusions**: This study uncovers the biomechanical impacts of meniscal injuries, demonstrating how the ACL compensates for various meniscus conditions. In contrast, transplantation and repair conditions only slightly increase the stress on the ACL, putting much less strain on the ACL and supporting structures of the knee joint than an unrepaired tear.

## 1. Introduction

The anterior cruciate ligament (ACL) is a prominent stabilizer. It serves as the primary restraint for anterior tibial translation, restraining the anterior motion of the tibia about the knee [1,2,3]. Within the knee, the ACL works with other structures that provide secondary support, such as the menisci, fortifying the knee’s integrity. The ACL comprises many fibers, making it strong and stiff. However, its particular architecture renders it prone to injury [4,5]. While ACL tears are the most common ligament injury in the United States, they can be severely debilitating. Symptoms encompass a range of possibilities: severe pain exacerbated by weight bearing, swelling of the knee, instability in the knee, discomfort while walking, limited knee movement, and the feeling that the knee is giving out are among the most common symptoms [6,7]. Surgical repair is often indicated due to the impacts on daily life and discomfort caused by tears in the ACL. It has been shown to reduce subsequent instability problems and meniscus injuries compared to those who do not receive surgical treatment [7].

Tears to the ACL, both vertical and horizontal, have been the focus of many studies [6,7], and limited attention has been paid to the effect on the ACL when compensating for injuries to the meniscus. Horizontal cleavage tears (HCTs) are one of the most common types of injuries of the meniscus. An HCT is a tear in the meniscus that runs parallel to the tibial plateau and travels through the mid-substance of the meniscus [8]. Small HCTs can go unnoticed, but when large, they can present with pain during activity, decreased range of motion, and stiffness.

We are offered a choice of repair method. Each option has its set of consequences and benefits to consider. Biomechanical studies have shown that preserving the meniscus is essential for mitigating degenerative changes in the knee after sustaining a meniscus tear [8,9].

It is important to note that clinical diagnosis of an anterior cruciate ligament (ACL) tear is still based on three standard tests: anterior drawer, pivot shift, and Lachman. A new lever test, first described by Lelli et al. in 2014 and further highlighted by Bucher et al. in 2022 through a study to show how to determine the sensitivity of the lever sign test for the clinical diagnosis of ACL tears, had some potential [10,11]. When used in the primary care setting of patients with acute knee injuries, Bucher et al. further calculated the positive predictive value (PPV) of the lever sign test by comparing it to the PPV of the Lachman test and its sensitivity [11]. A meta-analysis aimed at assessing the lever sign test’s diagnostic efficacy for ACL injuries was also conducted by Hu et al. in 2024, where the lever sign test was found to be a reliable diagnostic modality for ACL injuries [12]. Still, it should be used with other diagnostic tests to increase the accuracy of the diagnosis. Hesmerg et al. in 2024 further supported the high fidelity of the lever test in their systematic review [13].

Krakowski et al. 2019 provided a comparative study examining the diagnostic accuracy of physical examination and MRI in the most common knee injuries. Their finding supports that the McMurray and Apley tests for meniscal lesions seem the most appropriate in daily practice. A combination of lever signs, pivot shifts (PSs), and Lachman tests showed the best sensitivity and specificity in detecting ACL deficiency and was superior to MRI [14]. Krakowski et al. further discussed how intraarticular knee joint lesions commonly affect menisci and ligaments. If untreated and undiagnosed, the lesions progress over time and damage cartilage [15]. This highlights the importance of examining ACL injuries in the presence of meniscus in different conditions.

Previous studies have shown that injuries to knee structures and meniscus mainly result in changes in the femur tibia contact area [16]. In this study, we sought to investigate the effect HCTs have on the ACL. We will analyze these effects through 3D finite element analysis model simulations. The finite element analysis model was developed to replicate the experimental conditions on human cadaveric knees. This comprehensive model encompassed four scenarios, each representing distinct meniscal conditions. From these experimental conditions, the finite element analysis model was validated and subsequently used to evaluate the ACL under the same conditions.

## 2. Method and Materials

Seven fresh-frozen human cadaveric knees were subjected to a thawing process, followed by a meticulous arthrotomy examination to inspect for arthritis or potential damage to the meniscus or ligaments. The donor pool consisted of six male donors and one female donor, with an average age of 78 (ranging from 46 to 98).

An orthopedic surgeon (J.K.) with over 15 years of experience specializing in knee surgery carefully evaluated each specimen before inclusion. All specimens were procured by a donating organization (Science Care) and prepared by removing the skin and muscle attachments while preserving the patella, quadriceps tendon, and patellar tendon for specimen mounting.

The knee preparation process involved meticulously removing surrounding muscle tissue and the patella. Following the preservation of the posterior meniscocapsular attachment, utmost care was taken to maintain the integrity of essential supporting ligaments, including the anterior cruciate ligament (ACL), posterior cruciate ligament (PCL), medial collateral ligament (MCL), and lateral collateral ligament (LCL). Subsequently, anterior and posterior submeniscal arthrotomies were conducted to enable the insertion of sensors (Tekscan, Inc., Norwood, MA, USA) for data capture.

An oblique osteotomy of the medial femoral condyle was meticulously performed by initiating the cut from the posterior aspect of the condyle and extending it anteriorly using a saw blade. This osteotomy facilitated access to the meniscus and preserved the structural integrity of crucial ligaments, encompassing the ACL, PCL, MCL, and LCL. To guarantee both stability and precision throughout the testing phase, we firmly anchored the osteotomy using two screws positioned perpendicular to the osteotomy site.

The experimental conditions for the human cadaveric knees comprised four specific scenarios: intact, partial horizontal cleavage tear (HCT), partial meniscectomy, and partial transplantation. In the intact meniscus scenario, the meniscus remained in its natural, undamaged state without any tears or surgical interventions. The HCT was intentionally introduced in the posterior horn of the meniscus, using a number-11 blade. This cleavage tear was precisely situated approximately 2 cm away from the meniscus root and extended up to the attachment point of the root to the MCL in the posterior region of the meniscus [17]. In the third testing condition, the meniscus’ superior and inferior portions were excised using an arthroscopic biter toward the capsule, leaving the peripheral rim intact. In the partial transplantation condition, a 2-0 Ticron suture (Smith and Nephew, London, UK) was utilized with a four-vertical mattress suture technique for the allograft transplantation.

### 2.1. Mechanical Testing and Sensor Placement

The tibia and femur were firmly attached to a specially designed testing apparatus using cylindrical clamps. The tibia and femur were secured in custom fixtures to prevent sliding under load using eight M8 −1.25 × 80 mm^2^ bolts. The femur was connected to a uniaxial load frame (MTS 30/G machine, Portland, OR, USA) for axial loading from 0 to 600 N at full extension or 30 degrees of flexion, replicating typical knee positions during human gait. To simulate real-world conditions, an axial load of 800 N was applied three times to mimic the full-body load during single-leg standing, ensuring consistent load on the testing rig. The crosshead speed was 1 mm/s, and the machine’s data acquisition rate was 10 Hz. Contact pressures between the femoral and tibial condyles were measured using flexible digital pressure sensors inserted into the medial and lateral knee joint compartments (Tekscan model 4000).

### 2.2. Statistical Analysis

Given the inherent anatomical variability among individual cadaveric specimens, the Friedman test was performed after conducting a Shapiro–Wilk normality test to investigate the peak contact pressures/stress and contact area on both femoral condyles, applying a significance threshold of *p* ≤ 0.05. The Wilcoxon signed-rank test with Bonferroni correction was performed to examine variations in meniscal conditions at the knee’s full extension and flexion angles.

### 2.3. Finite Element Modeling

A 3D finite element model of the knee joint was created using Ansys software (version 2022, Ansys, Inc., Canonsburg, PA, USA). The model was based on a scan of a cadaveric knee joint using a micro-computed tomography (Micro-CT) system. The images from the scan were imported into the software to build a 3D representation of the knee joint. The bones’ surfaces were identified and separated using Mimics software (version 24.0, Materialise, Leuven, Belgium) and transformed into a surface mesh. Cartilage surfaces were established with SpaceClaim software (version 2022, ANSYS, Inc., Canonsburg, PA, USA) by extending a layer from the bone surface using the skin surface feature. The resulting cartilage layer was meshed and integrated into the model. The mesh was refined to ensure reasonable element aspect ratios and sizes were attained, resulting in a final mesh of approximately 88,000 elements. Convergence testing was performed to validate the model.

**Figure 1:**
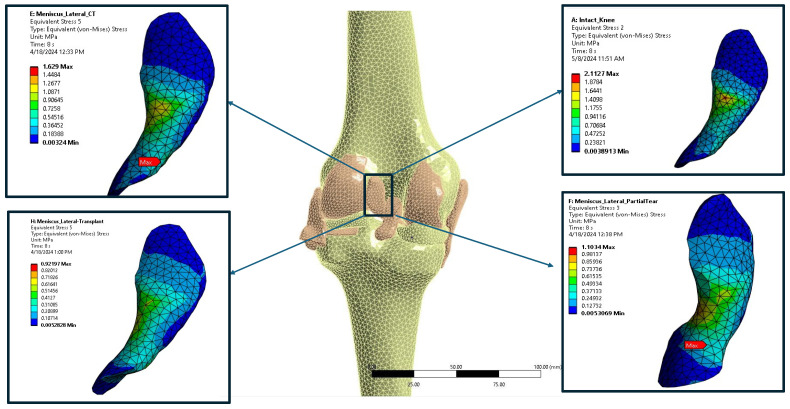
FEA stress mapping of the ACL under the four testing conditions (intact, horizontal cleavage tear (HCT), partial meniscectomy, and partial transplantation) at 0 knee flexion.

The finite element analysis (FEA) model was created to simulate the conditions of human cadaveric knees in an experiment. This detailed model included four main scenarios, each representing different meniscal conditions (Figure 1). A 2 cm long horizontal tear was found in the body of the medial meniscus, covering 80% of its width. A part of the meniscus was removed for the partial meniscectomy to eliminate the affected area. After that, the transplantation involved using the same removed part of the meniscus but with different material properties. These specific material properties are listed in Table 1.

## 3. Results

### 3.1. Mechanical Testing

At 0 degrees of knee flexion, our analysis revealed significant variations in contact pressure of the meniscus among different meniscal conditions when compared to the intact contact pressure, which measured 2684 ± 191.02 kPa, and the contact area, which measured 690.3 ± 63.00. Notably, contact pressure exhibited a significant increase in cases of HCT (2861 ± 96.81 kPa) and partial meniscectomy (2884 ± 106.1 kPa) procedures (*p* = 0.008 and *p* = 0.028, respectively). In contrast, contact pressure experienced a decrease when partial transplantation was performed. This reduction was statistically significant (*p* = 0.051 and *p* = 0.002) compared to the contact pressure after HCT and meniscectomy. The relationship between contact area and contact pressure revealed discernible disproportionality. Despite the contact area for HCT measuring 630.2 ± 118.4, which experienced a significant decrease (*p* = 0.028), the contact area following partial meniscectomy, quantified at 574.3 ± 42.43, exhibited a statistically significant reduction (*p* = 0.06) when compared to the intact area. The contact pressure pattern transitioned from the posteromedial to the anterolateral aspect, progressing from an intact state to a partial tear and subsequent meniscectomy.

**Figure 2:**
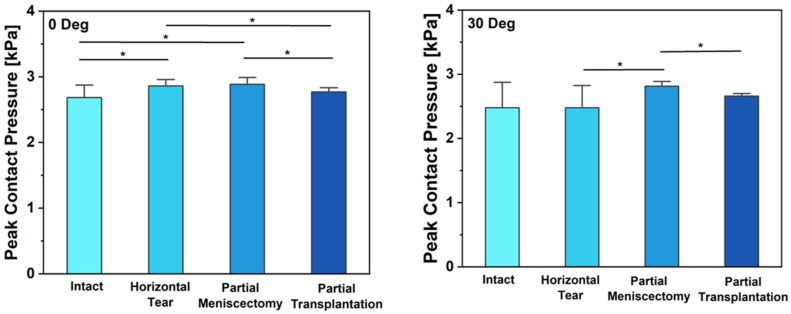
The peak contact pressure (mean ± standard deviation) of the meniscus was measured across the sensor under the four testing conditions (intact, horizontal cleavage tear (HCT), partial meniscectomy, and partial transplantation) at 0 and 30 degrees of knee flexion. Significant differences between the groups are denoted with an asterisk (*p* < 0.05).

At a knee flexion angle of 30 degrees, our observations revealed a pattern in contact pressure and contact area comparable to what was identified at 0 degrees. However, it is noteworthy that the significance levels exhibited variation in this context. The contact pressure for HCT (2479 ± 345.2 kPa) and partial tear (2814 ± 72.79 kPa) did not significantly differ from the contact pressure of the intact condition (2477 ± 395.4 kPa) (Figure 2). However, a significant decrease in contact pressure was observed after partial transplantation (*p* = 0.008). The contact area for partial meniscectomy (558.5 ± 48.25) and partial transplantation (604.0 ± 40.0) displayed a reduction when contrasted with the intact area (673.5 ± 19.19). The fluctuations observed primarily pertained to the magnitude of pressure rather than significant alterations in its distribution (Figure 2).

### 3.2. Finite Element Analysis

A finite element method (FEM) is a mathematical technique that allows for predictive calculations of a specific object’s behavior to be made through the process of finite element analysis (FEA). These techniques will enable us to obtain predictive measurements through a simulation of things that we may not be able to measure directly in experiments.

In this study, we conducted simulations to investigate the effects of HCT of the meniscus, partial meniscectomy, and partial transplantation on the mechanical behavior of the knee joint. The contact area and contact pressure measurements were meticulously performed across these scenarios. Subsequently, the findings were validated by employing a finite element analysis (FEA) model derived from micro-CT-based images.

**Figure 3:**
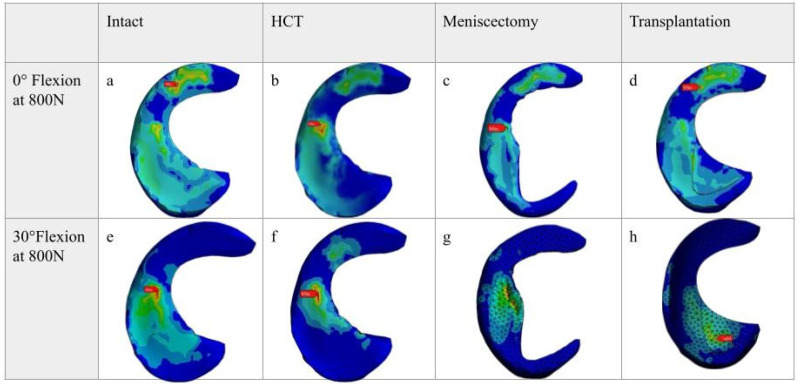
Pressure contour mapping of the meniscus across the four testing conditions (intact, horizontal cleavage tear (HCT), partial meniscectomy, and partial transplantation) at 0 (**a**–**d**) and 30 degrees (**e**–**h**) of knee flexion at 800 N applied force.

The stress changes observed in each meniscus case are similar to the findings from our experimental study, although with different numerical values (Table 2). In all cases, the highest stress values were observed within the meniscus body (Figure 3). During full extension (Figure 3a–d), the von Mises stress in the intact model was measured at 1.61 MPa. The stress increased to 2.25 MPa, a 39.75% increase, in the case of HCT and peaked at 2.85 MPa, a significant 77.01% increase from the intact state in the case of meniscectomy. After transplantation, the maximum stress levels increased by 26.71% compared to the intact model but decreased by 28.42% compared to the meniscectomy model. Conversely, during a 30-degree flexion motion (Figure 3e–h), the von Mises stress reached 2.32 MPa, representing a substantial 44.09% rise compared to the full extension scenario, with stress values predominantly concentrated within the meniscus body. HCT (2.76 MPa) and meniscectomy (2.99 MPa) exhibited similar stress levels, with respective increments of 19.39% and 28.87% from the intact state. Following transplantation, stress values decreased to 1.91 MPa, indicating a 17.67% reduction compared to the intact model and a 45.02% decrease from the HCT scenario.

**Figure 4:**
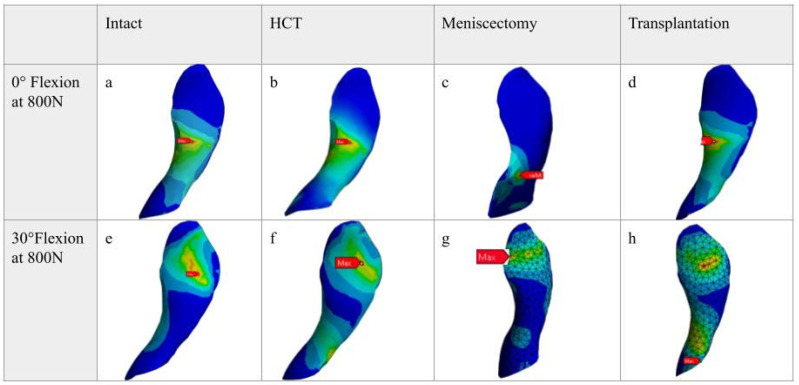
Stress contour mapping of the ACL across the four testing conditions (intact, horizontal cleavage tear (HCT), partial meniscectomy, and partial transplantation) at 0 (**a**–**d**) and 30 degrees (**e**–**h**) of knee flexion at 800 N applied force. The 4 conditions for 0 and 30 degrees are labelled.

Comparing the human knee experimental data with the FEA model for each testing condition validates the model (Table 3). There was no statistically significant difference in the data when comparing the FEA model to the experimental conditions. We had to employ the FEA model we created to assess the impact on the ACL during the test conditions as we subjected the meniscus to compressive loads. This allowed us to observe the changes to ACL stress conditions for each experimental condition. The 0-degree Flexion at 800 N are shown in Figure 4a–d and the 30-degree flexion are respectively shown in Figure 4e–h. 

At 0 and 30 degrees of flexion, our analysis revealed significant variations in stress on the ACL among the different meniscus conditions. The stress on the ACL with the meniscus intact measured an average of 0.607 MPa (SD = 0.700 MPa). ACL stress exhibited a significant increase in the meniscectomy condition (Avg: 1.634 MPa, SD = 1.884 MPa), with a *p*-value of 0.049 compared to the intact condition. Unlike meniscectomy, the ACL stress experienced a decrease when partial transplantation was performed (Avg: 0.937 MPa, SD = 0.927 MPa). Although other comparisons did not reach statistical significance, the trend suggests that meniscectomy increases ACL stress, while partial transplantation helps reduce it. The stress on the ACL in the HCT condition was also elevated (Avg: 0.872 MPa, SD = 0.900 MPa), though not as significantly as in the meniscectomy condition.

At 30 degrees of flexion, our analysis demonstrated a similar pattern in ACL stress to what was identified at 0 degrees. Of note, the significance levels exhibited variation at 30 degrees. 

Analyzing stress distribution on the anterior cruciate ligament (ACL) at these specific angles is particularly important because they reflect common functional positions during athletic movements. Focusing on these angles also allows for a larger contact area between the femoral condyles and the tibial plateau, influencing stability and load distribution across the joint. By understanding how the ACL and surrounding structures respond under these varying conditions, we can better predict potential injury risks and develop targeted rehabilitation strategies.

## 4. Discussion

This study aims to extensively evaluate the stress transmitted to the ACL in response to dynamic physiological loads applied to different changes undergone by the medial meniscus. Our specific focus is the ACL response and its changes in stress and strain consequent to HCTs, partial meniscectomy, and partial transplantation procedures.

Our findings indicate substantial variability in ACL contact pressure and stress in the various testing conditions. When measuring the stress on the ACL in the presence of horizontal cleavage tears (HCTs), the results showed increased contact pressures and diminished joint stability, as evidenced by increased ACL stress attributed to compensatory mechanisms. In the scenario of a partial meniscectomy, the results showed increased contact pressure and diminished joint stability. Conversely, testing in the partial transplantation setting exhibited a mitigating effect. The transplanted meniscus was able to maintain joint mechanics closer to intact conditions, minimize maintained joint mechanics closer to intact conditions, and reduce alterations in ACL forces. In these trends, significant increases in ACL forces were observed in the HCT and partial meniscectomy conditions, contrasting with the minimal changes observed in transplantation scenarios. It is essential to highlight some limitations of this study, such as the age of the cadaver’s knee. It is uncertain whether younger specimens would have shown improved results in the experiments or if their inclusion would have led to further finite element analysis (FEA) simulations. Conducting a correlation study would be beneficial for better validating the proposed model.

These findings align with prior research that assessed contact pressure following meniscectomy, showing decreased contact area and increased joint instability and contact pressure compared to the intact conditions at 0 degrees and 30 degrees of knee flexion [16,17,18]. Prior studies by Sunjung, Amirouche, et al. in 2024 demonstrated the impact of defect size, location, and alignment on knee joint contact pressures [17,19,20,21]. Intervening promptly with defects exceeding 3 mm is crucial, as significant stress levels manifest beyond this threshold. Significant increases in contact pressures were noted with larger defect sizes, particularly between 3 and 10 mm at full extension. FEA validated increasing contact pressures up to 7 mm defect size, beyond which pressures stabilized or slightly decreased. These findings inform our understanding of the biomechanical implications of OCDs. Partial tear repairs were addressed with allographs and showed that contact pressure for various loading was similar to healthy knees. This finding, however, is limited to horizontal tears and requires clinical data for validation.

This study highlights the importance of the ACL response to different meniscus pathologies and repairs.

Many studies have found that ACL tears correlate with meniscal injury. Meunier et al. [7] investigated the long-term outcomes of 100 patients after 15 years of having surgical or non-surgical treatment of an ACL tear. The results showed a significant increase in meniscus injuries in patients who were initially treated non-surgically compared to those treated with surgical intervention. A large 1⁄3 of the non-surgically treated patients in Meunier’s study later underwent ACL reconstruction due to the continuous instability and secondary meniscus tear developed. Meunier’s study and many others support the hypothesis that early stabilization of the knee after an ACL injury is helpful for long-term outcomes, and this poses the question of whether the inverse was true. From our study, we can conclude that injuries to the meniscus do indeed increase the stress and tension on the ACL and can predispose it to injury and further wear and tear damage. That being said, longitudinal studies should be conducted to assess the overall outcomes and differences in ACL integrity between groups receiving surgical meniscus repair and those who do not.

Despite the findings in these studies and our own, other questions remain. How could such FEA models be extended to simulate more dynamic motion analysis scenarios of various deficiencies in meniscus and ACL? How can we capture more realistic imaging data using DIC techniques to capture strains and stresses of the injured knee? Does early stabilization and repair after a meniscus injury significantly reduce the risk of secondary ACL injury? Is it more beneficial to surgically intervene or treat in more conservative, non-surgical ways? We know that surgical intervention and repair of the meniscus reduces stress on the ACL. It is still unknown whether or not it significantly reduces the long-term risk of secondary ACL injury. Continuous research and patient follow-ups may answer these questions, and patient clinical outcomes will help guide the surgeon’s future decisions.

## 5. Conclusions

The experimental and computational results validate stress patterns on the ACL that are associated with meniscus injuries, explicitly addressing the use of HCT, meniscectomy, and transplant procedures as potential solutions. The primary findings of the study are summarized as follows:

1. HCT and partial meniscectomy significantly increased stress on the ACL and decreased contact area compared to the intact condition.
2. Transplantation with autograft may reduce contact pressure following meniscectomy and increase contact area.
3. Stress and contact area variations are observed with different knee extension and flexion degrees.
4. The FEA model appropriately demonstrates similar patterns to the experimental results; however, variations may occur depending on material properties and the accuracy of modeling details.

These findings offer significant insights into the biomechanical implications of meniscus injuries on the ACL. Future studies may explore innovative surgical techniques or materials to preserve the meniscal function better, improving the amount of stress on the ACL after a meniscal injury. Additionally, there is potential for refining biomechanical models used to simulate meniscus injuries by integrating more comprehensive anatomical and material properties into finite element analysis (FEA) models, thereby improving their accuracy and predictive capacity.

## Figures and Tables

**Table 1:** Material properties with annotated references are used in the proposed model.

Component	Modulus of Elasticity (MPa)	Poisson Ratio
Cortical Bone [16]	17,900	0.36
Articular Cartilage [16]	15	0.475
ACL [17]	366	0.45
PCL [17]	131.5	0.45
Meniscus [17]	120	0.45
Allograft Meniscus [17]	110	0.49
MCL and LCL [17]	400	0.45

**Table 2:** The meniscus’ maximum stress from Finite Element Analysis was determined under the four testing conditions: intact, horizontal cleavage tear (HCT), partial meniscectomy, and partial transplantation.

	Intact 0° (Mpa)	Intact 30° (Mpa)	HCT 0° (Mpa)	HCT 30° (Mpa)	Meniscectomy 0° (Mpa)	Meniscectomy 30° (Mpa)	Transplant 0° (Mpa)	Transplant 30° (Mpa)
200 N	0.02285	0.080199	0.034295	0.045505	0.038426	0.040716	0.023877	0.37298
400 N	0.21785	0.41907	0.3147	0.4141	0.38866	0.38959	0.24642	0.3186
800 N	1.611	2.4661	2.24587	2.9964	2.8529	2.9864	2.0389	0.9097

**Table 3:** The ACL’s maximum stress is due to the finite element analysis under the four testing conditions: intact, horizontal cleavage tear (HCT), partial meniscectomy, and partial transplantation.

	Intact 0° (Mpa)	Intact 30° (Mpa)	HCT 0° (Mpa)	HCT 30° (Mpa)	Meniscectomy 0° (Mpa)	Meniscectomy 30° (Mpa)	Transplant 0° (Mpa)	Transplant 30° (Mpa)
200 N	0.059588	0.10544	0.059686	0.080849	0.048666	0.113	0.063123	0.37298
400 N	0.39257	0.33579	0.38795	0.45244	0.61784	0.60704	0.41833	0.3186
800 N	2.1127	0.63775	2.0526	2.2008	4.9583	3.4595	2.4565	1.9097

## Data Availability

FEA data supporting reported results can be available by corresponding author.
